# Oxygen Atom Stabilization by a Main-Group Lewis Acid: Observation and Characterization of an OBeF_2_ Complex with a Triplet Ground State

**DOI:** 10.1021/jacs.4c07079

**Published:** 2024-08-15

**Authors:** Guohai Deng, Marc Reimann, Deniz Meyer, Xiya Xia, Martin Kaupp, Sebastian Riedel

**Affiliations:** † Institut für Chemie und Biochemie−Anorganische Chemie, Freie Universität Berlin, Fabeckstrasse 34/36, Berlin 14195, Germany; ‡ Institut für Chemie, Theoretische Chemie/Quantenchemie, Technische Universität Berlin, Sekr. C7, Strasse des 17. Juni 135, Berlin 10623, Germany

## Abstract

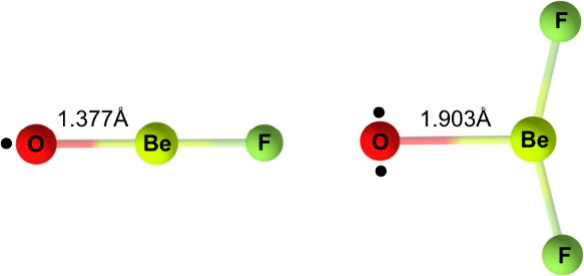

Terminal oxygen radicals involving p- and d-block atoms are quite common, but s-block compounds with an oxygen radical character remain rare. Here, we report that alkaline-earth metal beryllium atoms react with OF_2_ to form the oxygen beryllium fluorides OBeF and OBeF_2_. These species are characterized by matrix-isolation infrared spectroscopy with isotopic substitution and quantum-chemical calculations. The linear molecule OBeF has a ^2^Π ground state with an oxyl radical character. The ^3^A_2_ (*C*_2*v*_) ground state of OBeF_2_ represents the unusual case of a triplet oxygen atom stabilized by a relatively weak interaction by the Lewis acidic BeF_2_. The interaction involves both a donor component from oxygen to empty Be orbitals and a back-bonding contribution from fluorine substituents toward oxygen.

## Introduction

**Scheme 1:**
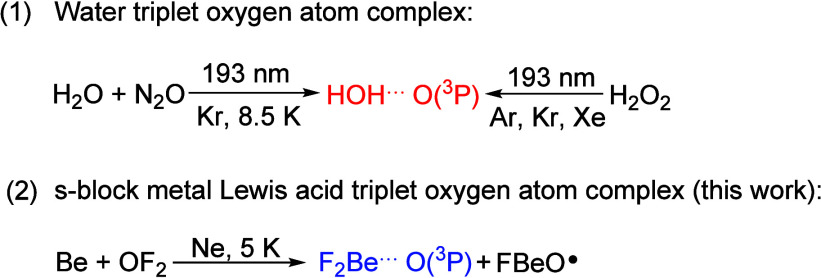
(1) A Directly Observed Triplet Oxygen Atom Complex of Water^12,13^ and (2) the Oxygen Atom Complex of the Main-Group Lewis Acid BeF_2_ (Present Work)

Metal–oxygen interactions play a critical role in several important areas of chemistry, such as electrochemistry,^1,2^ semiconductor devices,^3^ astrochemistry,^4^ and heterogeneous catalysis^5,6^ to initiate, for example, oxidation reactions. In such heterogeneous reactions, the catalytic activity of the material is strongly dependent on its ability to activate molecular oxygen and moderately bind the active oxygen species to the metal.^7,8^ Therefore, middle and late transition metals such as Ru, Rh, Ir, Pd, and Pt are usually promising candidates because the d*-*orbitals are almost full of valence electrons, the terminal oxo ligand is destabilized, and the complexes become more reactive.^9−11^ In most cases, however, dissociated oxygen atoms tend to become chemisorbed or transformed into other species (e.g., oxyl or hydroxyl radicals). This leads to a wide variety of oxygen coordination motifs, such as terminal, bridged, 3-fold, or even higher coordinated O species, of which only a few exhibit catalytic activity. An ideal active metal would have only one active coordination motif, in which the oxygen species are moderately bound in a reactive electronic configuration. This may be achieved by a saturated metal center, which still allows further coordination and exhibits Lewis acidic behavior. In any case, equilibria between bound and gas-phase oxygen atoms may be important in such processes. Weaker interactions with surfaces intermediate between chemisorption and physisorption have been found, for example, for adsorption of oxygen atoms on water ice in an astrochemical context, where quantum-chemical calculations suggest an interaction with water hydrogen atoms with a binding energy of the order of about 12 kJ mol^–1^.^4^ Such water oxygen atom interactions via hydrogen bonding have also been investigated by matrix-isolation experiments in which the triplet oxygen atom complex of a water molecule was produced by photolysis of H_2_O/N_2_O mixtures or H_2_O_2_ at cryogenic temperatures (Scheme 1, (1)).^12,13^ Another study showed that photochemically generated oxygen atoms in, e.g., water can cross a semipermeable nanocapsule barrier and react with the separated reactant. Based on these observations, a mechanism based on a freely diffusing O(^3^P) atom was assumed.^14^

Here, we report on the moderate stabilization of a triplet oxygen atom in a donor–acceptor complex with BeF_2_ under low-temperature matrix-isolation conditions (Scheme 1, (2)). Beryllium, the lightest group 2 element, has been considered previously in the context of stabilizing unusual species, largely due to its high ionization energy and small atomic radius. As a consequence, its bonding interactions often feature considerable covalent character, unlike the heavier alkaline-earth metals.^15^ In the context of group 2 metal oxides, beryllium oxide species are among the most studied because of their unique bonding situation. The diatomic beryllium monoxide molecule BeO was found to exhibit an extraordinarily strong Lewis acid character and was found to be able to form the noble gas complexes NgBeO (Ng = He–Xe).^16−19^ Higher beryllium oxide species, such as OBeO, Be(O_2_), Be(O_2_)_2_, OBeOOO, OBe(O_3_), and Be(O_3_)_2_, have also been synthesized and characterized in solid noble gas matrices, with notable Be–Ng interactions.^20,21^ In addition to the neutral complexes, beryllium oxide anion BeO^–^ and dication BeO_2_^+^ are produced in the gas phase and have been detected by photodetachment spectroscopy^22^ and mass spectroscopy,^23^ respectively. Moreover, the nature of the chemical bond in the simplest terminal beryllium oxo compound HBeO has been investigated through single- and multi-reference correlation methods.^24^ However, this mode of simple terminal oxo compound L_*n*_BeO (L = any ligand except oxygen) is completely unknown experimentally.

We previously succeeded in using OF_2_ molecules and metal atoms for the synthesis of metal oxyfluoride molecules under cryogenic conditions in rare gas matrices.^10,25−27^ Therefore, the formation of beryllium oxyfluoride molecules may also be possible. In this work, we report for the first time the preparation of molecular oxygen fluorides of beryllium (OBeF and the OBeF_2_ complex) in solid neon matrices. They are identified by matrix-isolation infrared spectroscopy supported by isotope labeling of ^18^OF_2_ and state-of-the-art quantum-chemical calculations. This investigation provides insight into the nature of the unusual coordinative beryllium–oxygen bond in OBeF_2_ and an experimentally verified terminal oxyl radical in the case of OBeF.

## Results and Discussion

**Figure 1:**
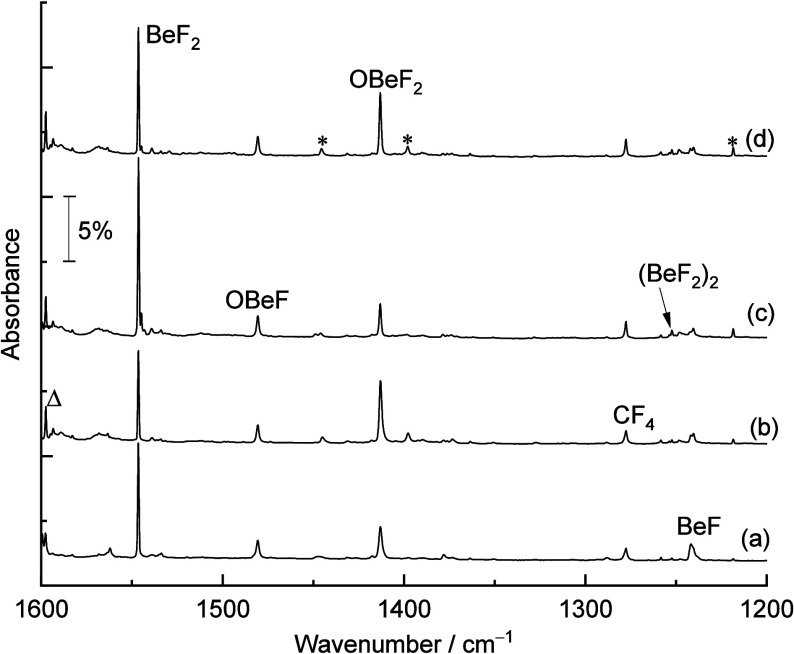
Infrared spectra in the 1600–1200 cm^–1^ region from codeposition of laser-ablated Be atoms with 0.05% OF_2_ in neon. (a) After 30 min of sample deposition, (b) after annealing to 10 K, (c) after 10 min of full arc (λ > 220 nm) irradiation, and (d) after annealing to 10 K. The Δ denotes water absorption. The bands of unidentified species (*) are labeled.

**Figure 2:**
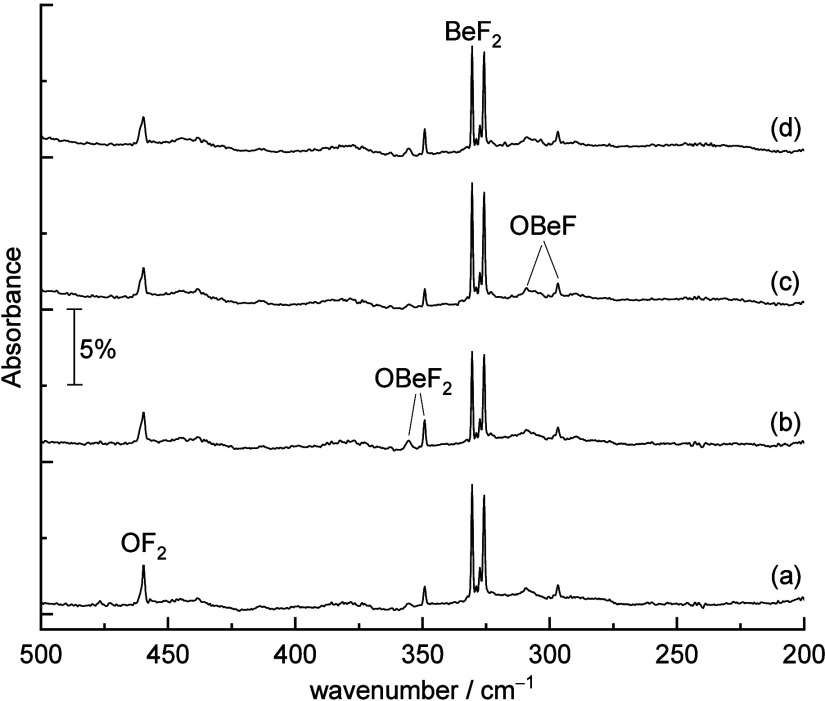
Far-infrared spectra, recorded via a bolometer, in the 500–200 cm^–1^ region from codeposition of laser-ablated Be atoms with 0.05% OF_2_ in neon. (a) After 30 min of sample deposition, (b) after annealing to 10 K, (c) after 10 min of full arc (λ > 220 nm) irradiation, and (d) after annealing to 10 K.

The beryllium oxygen fluoride species were produced via the reactions of laser-ablated beryllium atoms and OF_2_ molecules in solid neon. The infrared spectra in the 1600–1200 and 500–200 cm^–1^ regions from the codeposition of Be atoms with 0.05% OF_2_ in neon are shown in Figures 1 and 2, respectively. The common fluorine impurity bands for CF_4_^28^ at 1277.7 cm^–1^ and absorption bands at 1546.5, 1252.0, and 1241.6 cm^–1^ were detected in the reaction of Be with F_2_ under excess neon, which have previously been assigned to BeF_2_, (BeF_2_)_2_, and BeF, respectively (Figure 1).^29^ In addition, two lower bands at 330.5 and 325.8 cm^–1^ of BeF_2_ were also detected by our bolometer experiments (Figure 2). The absorption bands due to beryllium oxide are hardly observed. In addition to these known fluoride molecules, new product absorption bands were observed at 1480.6, 1423.1, 355.5, 349.3, 309.3, and 296.7 cm^–1^ in the present work. The experiments were repeated under the same conditions using an isotopically labeled ^18^OF_2_ sample to help product identification based on isotope shifts. The IR spectra in the selected region are shown in Figures S1–S5 in the Supporting Information. All observed band positions are summarized in Table 1.

**Table 1:** Experimentally Observed and Calculated Vibrational Frequencies (cm^–1^) and Intensities (km mol^–1^) for OBeF and OBeF_2_ at the CCSD(T*)-F12a/(aug)-cc-pVTZ-F12 Level

	exp.		calc.^a^		
species	ν(^16^O)	Δν(^16/18^O)	ν(^16^O)	Δν(^16/18^O)	assignment
OBeF	1480.6	7.2	1476.7 (318)	7.7	Be–F str.
	309.3	2.8	306.7 (193)	1.8	OBeF bending
	296.7	1.7	306.7 (193)	1.8	OBeF bending
OBeF_2_	1413.1	0.2	1419.9 (405)	0.1	asym. BeF_2_ str.
	355.5	0.6	355.6 (175)	0.2	Be–O str.^b^
	349.3	0.3	349.8 (197)	0.4	FBeF bending^b^

a The complete sets of vibrational frequencies are provided in Supporting Information, Tables S1 and S2. For the CCSD(T*)-F12a calculations, intensities were obtained from dipole surfaces obtained at the HF level.

b Tentative assignment due to the close energy of two vibrations.

The new bands at 1423.1, 355.5, and 349.3 cm^–1^ generated in the reactions of Be and OF_2_ were not detected in the experiments of Be reacted with F_2_ or O_2_.^20,21,29^ This set of bands appeared upon sample deposition and increased on annealing to 10 K but decreased upon irradiation with λ > 220 nm, and they are assigned to different vibrational modes of the oxygen–difluoride complex OBeF_2_. The first absorption band appeared in the Be–F stretching region and showed a very small Δν(^16/18^O) isotope shift of 0.2 cm^–1^ belonging to the antisymmetric F–Be–F stretching mode, which is slightly coupled to an oxygen atom motion. Due to the small mass of Be compared to F, the vibrational motion is more accurately described as the oscillation of a Be atom between the two fluorine atoms. Two other bands at lower wavenumbers at 355.5 and 349.3 cm^–1^ with oxygen isotope shifts of 0.6 and 0.3 cm^–1^ are tentatively assigned to the Be–O stretching and F–Be–F out-of-plane bending modes, respectively. Because the two vibrations are very close in energy, we cannot make a definite assignment.

The assignment of these bands to OBeF_2_ is also further supported by quantum-chemical calculations at the CCSD(T*)-F12a level of theory, which has recently been employed with great accuracy for vibrational frequencies of F_3_SiP and its isomers.^30^ Additional results at the DFT and CCSD(T) levels can be found in the Supporting Information. The calculated vibrational frequencies are shown in Table 1 and Table S1. The three predicted strongest bands of OBeF_2_ at 1419.9, 355.6, and 348.8 cm^–1^ with ^16/18^O isotope shifts of 0.1, 0.2, and 0.4 cm^–1^ are consistent with the experimentally observed values at 1423.1, 355.5, and 349.3 cm^–1^ (0.2, 0.6, and 0.3 cm^–1^). Regarding the assignment of the bands, it should be noted, however, that the relative position of the two lower-lying modes differs between harmonic frequencies and the employed anharmonic vibrational configuration interaction (VCI) treatment. Moreover, the calculated vibrational frequencies and intensities of the possible hypofluorite isomer FOBeF are shown in Table S3. The hypofluorite isomer shows different spectral signatures compared to those of the observed spectra, further supporting our assignment of the experimental bands. The UV–vis absorption spectrum of triplet OBeF_2_ was also calculated at the LR-SCS-CC2/aug-cc-pVQZ level. The results show a strong absorption band at around 200 nm. Estimating the effects of higher-order excitations at the LR-CCSDT/aug-cc-pVDZ level shifts this band to around 220 nm (see Table S4 for details). This is perfectly consistent with the experimental observation that the IR bands of OBeF_2_ decreased under the irradiation with full arc light (λ > 220 nm).

The set of absorption bands at 1480.6, 309.3, and 296.7 cm^–1^ in the spectra belongs to another new product molecule and shows less variation on annealing and sample irradiation than the group of bands at 1423.1, 355.5, and 349.3 cm^–1^ mentioned above. All of the bands were observed after sample deposition, almost did not change upon annealing to 10 K, and slightly increased upon irradiation with λ > 220 nm. They can be attributed to the OBeF molecule. The absorption at 1480.6 cm^–1^ just below the BeF_2_ signal with an oxygen isotopic shift of 7.2 cm^–1^ belongs to the antisymmetric O–Be–F stretching mode, which is significantly higher than for the corresponding O–Be–O stretching mode of the diradical OBeO complex at 1413.4 cm^–1^.^31^ Another two lower bands at 309.3 and 296.7 cm^–1^ with relatively small oxygen isotopic shifts (2.8 and 1.7 cm^–1^) are ascribed to the O–Be–F bending modes, respectively. However, since the OBeF molecule is linear, these bending modes must be degenerate. Calculations at the CCSD(T*)-F12a level show this degeneracy and good agreement with one of the signals (at 309.3 cm^–1^).

In principle, the degeneracy can be lifted in one of two ways, either by coupling of the electronic state to rovibrational degrees of freedom (Renner–Teller effect) or symmetry breaking by interaction with the noble gas matrix. When considering the Renner–Teller effect, we obtain two bands (at 295 and 306 cm^–1^, respectively), in excellent agreement with the experimental results. However, our calculations predict two additional bands from this interaction at 387 and 399 cm^–1^, which cannot be seen in the experimental spectrum. Considering that the interaction with a neon atom of the matrix leads to similar results (Table S5), coordination of one neon atom to OBeF as a simple model is favorable by about 1 kJ mol^–1^ (calculated reaction enthalpy at 0 K). It results in a slight bending of the O–Be–F angle and lifts the degeneracy of the bending modes. Our calculations suggest that one of the resulting bending modes gives rise to the signal at 296.7 cm^–1^, while the other signal most likely overlaps with the BeF_2_ bands. While we cannot distinguish between the two mechanisms experimentally, we note that the bending mode of BeF_2_ is also split by a similar amount. As the ground state of BeF_2_ is nondegenerate, this splitting cannot be created by the Renner–Teller effect but is most likely due to interactions with the matrix. Similar interactions between CaF_2_ and noble gas atoms (Ne, Ar, Kr, and Xe) have been studied computationally in earlier work.^32^ We therefore suspect the interaction with the matrix to be the more likely origin of splitting in the OBeF band.

It should also be mentioned that computational modeling of linear molecules with a ^2^Π state by DFT methods may suggest a sizable splitting of the bending modes. This arises from an artificial symmetry breaking of the orbital-degenerate state by unrestricted single-determinant methods, which will model only one component of the degenerate Π state. While these two frequencies correspond well to the vibrational frequencies that can be used to calculate the Renner parameter (see Table S6 for details), these are not the physically occurring Renner–Teller-split frequencies! In its heart, this is a technical problem as it is created by typical SCF solvers that will converge to close local minima. Using numerical second derivatives and ensuring that every displacement uses the lowest possible energy lead to the correct degenerate frequencies. A comparison between the erroneous approach and the correct results at the CCSD(T*)-F12a level is shown in Table S7.

**Figure 3:**
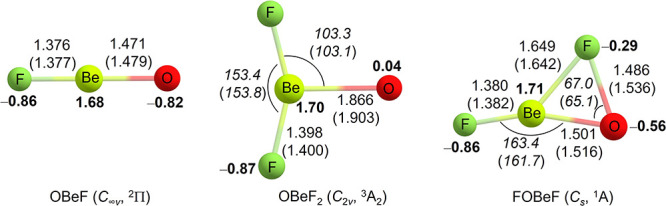
Calculated structures (bond lengths in Å and bond angles in degrees) and NPA charges (in bold, M06-2X level) of beryllium oxyfluorides at the M06-2X/def2-TZVPP and CCSD(T*)-F12a/aug-cc-pVTZ-F12 (in parentheses) levels.

To gain further insight into the structures and bonding in OBeF, OBeF_2_, and the unobserved hypofluorite FOBeF, quantum-chemical calculations have been performed using ab initio methods at the CCSD(T*)-F12a/(aug)-cc-pVTZ-F12 level and density functional theory (DFT) at the M06-2X/def2-TZVPP level. The optimized structures are listed in Figure 3. Both CCSD(T*)-F12a and M06-2X methods show linear structures with a doublet ground state for OBeF. At the CCSD(T*)-F12a level, the computed Be–F bond length in OBeF is 1.377 Å, slightly longer than that of free BeF (1.371 Å).^29^ The Be–O bond length is predicted to be 1.479 Å, indicating a Be–O single bond.^20,21^ Natural population analysis (NPA) suggests that the spin densities are mainly located at the O center (1.01 e, Figure S6), suggesting an oxyl radical character.

Optimized OBeF_2_ exhibits a ^3^A_2_ ground state with *C*_2*v*_ symmetry. The ^1^A’/*C*_s_ state of the FOBeF isomer is about 6.5 (M06-2X) or 2.2 kcal mol^–1^ (CCSD(T*)-F12a) higher in energy than this ^3^A_2_ state (Table 2). The calculated Be–O bond length at the CCSD(T*)-F12a level in OBeF_2_ is 1.903 Å, which is dramatically longer than the Be–O single bond length of 1.479 Å in OBeF. The very long bond is consistent with our experimental observation that the Be–O stretching mode is located at a lower wavenumber and has a very small oxygen isotope shift. The bond dissociation energy of OBeF_2_ for the loss of the oxygen ligand is 6.3 kcal mol^–1^ (CCSD(T*)-F12a) (Table 2), which is close to the dissociation energy of the O–O bond (about 6.5–7.4 kcal mol^–1^) in the HOON complex^33^ and in the ball park of the above-mentioned adsorption of oxygen atoms on ice surfaces in interstellar media.^4^ NPA charges at the M06-2X/def2-TZVPP level indicate that the bond between BeF_2_ and O is a weak donor–acceptor interaction without significant charge transfer: the computed NPA charge on oxygen is only 0.04 (Figure 3). The same analysis shows that the oxygen atom carries most of the spin density (1.99 e, see Figure S6). This species may be considered a triplet oxygen atom weakly stabilized by the interaction with a BeF_2_ Lewis acid. For comparison, the corresponding chlorido complexes OBeCl_2_ and ClOBeCl were also investigated computationally (Figure S7). Interestingly, here, singlet ClOBeCl is 41.9 kcal mol^–1^ below triplet OBeCl_2_ at the CCSD(T*)-F12a/(aug-)cc-pVTZ-F12 level (Table 2). This can be attributed to a much stronger Be–F than Be–OF bond, while the Be–Cl and Be–OCl binding energies are much more similar (the O–F bond is also slightly weaker than the O–Cl bond in the hypohalide complexes). Together, this leads to the slight favorability of the triplet BeX_2_–O complex compared to XOBeX with X = F (see Tables S8 and S9 in the Supporting Information for details). Notably, the Be–Cl interaction in triplet Cl_2_Be–O is even slightly more pronounced than with the fluoride (Table 2), but due to the above-mentioned factors, singlet ClOBeCl is much more stable.

**Table 2:** Calculated Reaction Energies of Beryllium Oxyfluorides and Oxychlorides (kcal mol^–1^) at the CCSD(T*)-F12a/(aug-)cc-pVTZ-F12 level^b^

	X = F		X = Cl	
reaction	Δ*E*	+ΔZPE	Δ*E*^a^	+ΔZPE^a^
Be + OX → OBeX	–201.5	–199.0	–147.9	–145.8
Be + OX_2_ → OBeX_2_	–219.5	–218.0	–130.1	–129.2^a^
OBeX_2_ → OBeX + X	59.6	58.9	19.9	20.0^a^
XOBeX → OBeX_2_	–2.2	–3.0	43.1	41.9^a^
OBeX_2_ → BeX_2_ + O	6.3	5.8	8.5	8.1^a^

a OBeCl_2_ is no minimum at this level, the zero-point vibrational corrections are therefore not perfectly valid.

b Δ*E* + ΔZPE corresponds to the reaction enthalpy at 0 K.

**Figure 4:**
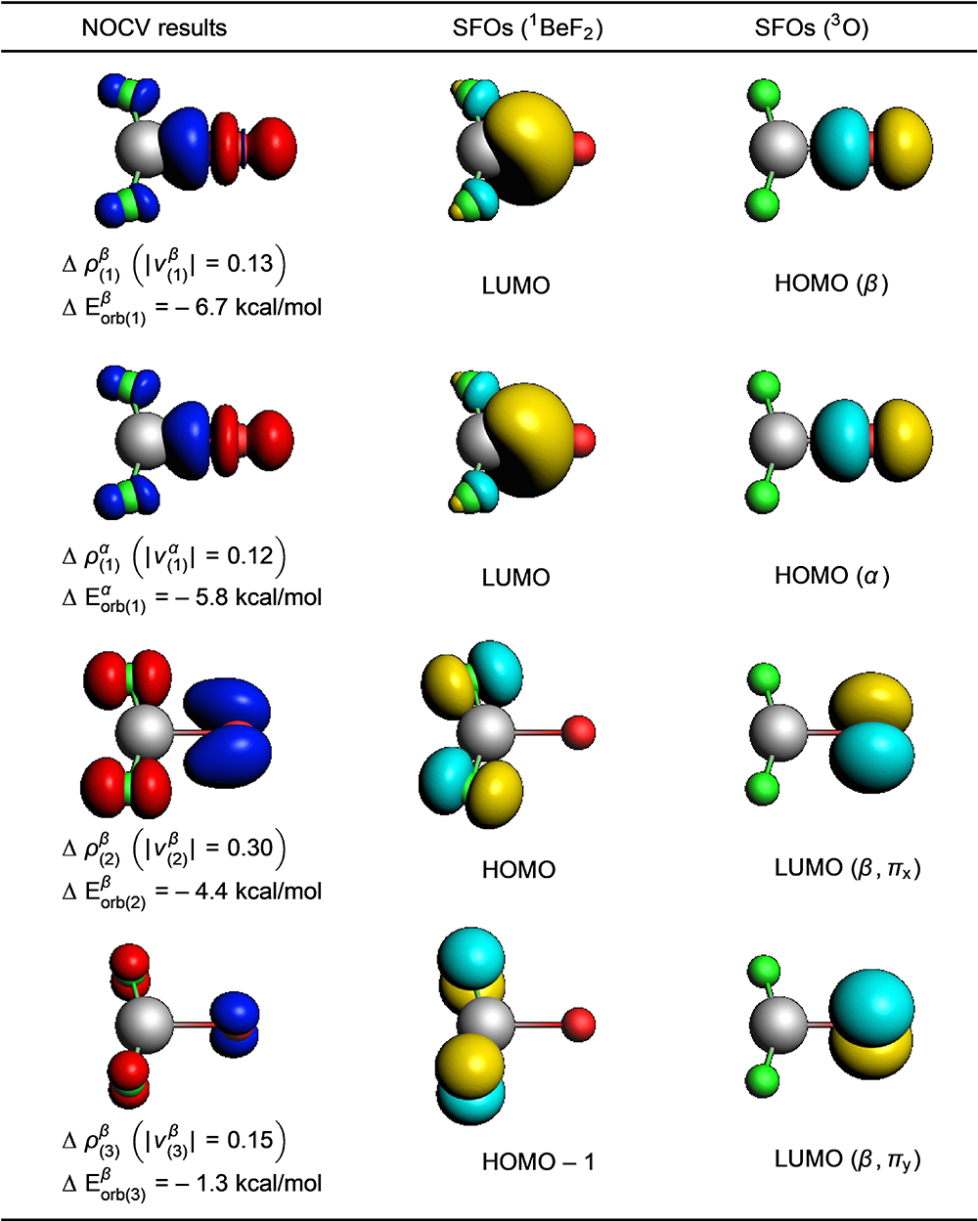
Shape of the four most important deformation densities from natural orbitals for chemical valence (NOCV) analyses for OBeF_2_ (^3^A_2_) as well as the respective symmetrized fragment orbitals (SFOs) at the BP86+D3(BJ)/TZ2P level. Isosurfaces of the deformation densities are shown at a value of 0.001, and charge flows from red to blue. Isosurfaces of the SFOs are shown at a value of 0.07.

For a closer analysis of the weak O–BeF_2_ interaction, we used an extended transition state and natural orbitals for chemical valence (ETS-NOCV) analysis.^34,35^ The results are shown in Figure 4. The interaction of the BeF_2_ fragment and the oxygen atom can be classified as a Lewis base coordination of the lone pair of a triplet oxygen atom to BeF_2_, forming a relatively weak σ donor–acceptor interaction. This is augmented by π back-donation from the lone pairs of the fluorine atoms to the partially filled orbitals at the oxygen atom.

Important reaction energies computed at the CCSD(T*)-F12a level, which may be used to estimate the formation mechanisms, are also listed in Table 2. IR-laser ablation generates excited beryllium atoms, which are expected to react directly with OF_2_ to yield OBeF_2_ during sample codeposition. The formation process is computed to be highly exothermic by −218.0 kcal mol^–1^. The product OBeF molecule is likely formed from the reaction of beryllium atoms with OF radicals (Be + OF → OBeF). The OF radicals might be generated by IR-laser- or beryllium-induced fluorine atom abstraction from OF_2_, the latter being suggested by detection of beryllium fluorides (BeF and BeF_2_). The calculated reaction energy for this reaction is −199.0 kcal mol^–1^.

## Conclusions

In summary, the beryllium oxyfluoride radicals OBeF and OBeF_2_ have been prepared via the reactions of beryllium atoms with OF_2_ molecules in solid neon and were characterized by matrix-isolation IR spectroscopy and isotopic substitution as well as electronic structure calculations.

The unusual OBeF_2_ has a triplet electronic ground state and may be described as a triplet oxygen atom stabilized by a relatively weak donor–acceptor interaction with BeF_2_, involving both a donor component from the oxygen lone pair to BeF_2_ and a back-bonding interaction from the fluorine lone pairs to the empty oxygen orbitals. The interaction is somewhat reminiscent of the adsorption of an oxygen atom on ice in an interstellar medium.

The linear OBeF molecule has a doublet ground state and exhibits an oxyl radical character. While similar beryllium species like HBeO have been examined before computationally, this seems to be the first experimentally verified case of such a species in beryllium chemistry.

## Supporting Information Available

Experimental infrared spectra, calculated structures, and vibrational frequencies (PDF)
