# Supplementary material for: Oxygen Atom Stabilization by a Main-Group Lewis Acid: Observation and Characterization of an OBeF2 Complex with a Triplet Ground State
Source: J Am Chem Soc. 2024 Aug 15;146(34):23972–7. doi: 10.1021/jacs.4c07079 (PMC11363016; doi:10.1021/jacs.4c07079)
Supplement: Supplementary file 1 — ja4c07079_si_001.pdf [file ja4c07079_si_001.pdf]

Supporting Information

Oxygen Atom Stabilization by a Main Group Lewis Acid: Observation and Characterization of a O-BeF_2 Complex with a Triplet Ground State

Guohai Deng†*, Marc Reimann‡, Deniz Meyer†, Xiya Xia†, Martin Kaupp‡*, Sebastian Riedel†*
† Institut für Chemie und Biochemie–Anorganische Chemie, Freie Universität Berlin, Fabeckstrasse 34/36, 14195 Berlin (Germany).
‡ Institut für Chemie, Theoretische Chemie/Quantenchemie, Sekr. C7, Technische Universität Berlin, Strasse des 17. Juni 135, 10623 Berlin (Germany)

Table of contents

Experimental and Theoretical Methods
Figure S1-S7
Table S1-S9
References
Reaction atomic coordinates

Experimental and Computational Methods

The experimental apparatus used for the laser ablation of beryllium atoms and their reaction with OF_2 in exceed neon gases, as well as their deposition at 5 K using a closed cycle helium cryostat (Sumitomo Heavy Industries, RDK-205D) inside a vacuum chamber has been described in more detail in our previous works.[1] $^{16/18}OF_2$ was synthesized by a known procedure using elemental fluorine and $^{16/18}OH_2$ dispersed in solid NaF.[2] The Nd:YAG laser fundamental (Continuum, Minilite II, 1064 nm, 10 Hz repetition rate with 10 ns pulse width) with a pulse energy of up to 55 mJ/cm² was focused onto the metal targets, which gave an energetic plasma beam reacting with OF_2 and spreading toward the cold rhodium-plated mirror. IR spectra were recorded in reflection using a Bruker Vertex 80v vacuum FTIR spectrometer equipped with a KBr beam splitter and a liquid nitrogen cooled MCT detector were used in the region of 5000–450 cm^{-1} and FIR multilayer mylar beam splitter with a liquid helium cooled bolometer was used in the region of 600–150 cm^{-1}. For each spectrum 200 scans at a resolution of 0.5 cm^{-1} were coadded. The matrix samples were annealed at different temperatures, and cooled back to 5 K for spectral acquisition. The selected samples were subjected to irradiation with a medium-pressure mercury arc streetlamp ($\lambda > 220$ nm).

Kohn-Sham density functional theory (DFT) calculations were performed at the M06-2X/def2-TZVPP[3] level with the Gaussian 16 program.[4] Natural population and natural bond orbital analyses[5] (NPA and NBO) were performed at the same level. Additional calculations were performed with the TURBOMOLE program package, version 7.7.1.[6] Extended transition-state analyses with natural orbitals for chemical valence (ETS-NOCV) were performed at the BP86+D3(BJ)/TZ2P level[7] using the ADF engine of the AMS software package (Release 2023.1).[8]

Coupled cluster calculations with single, double, and perturbative triple substitutions, CCSD(T), were carried out in the closed-shell (RHF-CCSD(T)) and partially spin-restricted open-shell (RHF-RCCSD(T)) formalism using default frozen core settings as implemented in the MOLPRO2022 software package.[9] To closely approach the basis set limit, explicitly correlated calculations were performed using the F12a approximation.[10] Explicit correlation effects on the perturbative triples were estimated by scaling the (T) contribution as:

$$\Delta Ecorr(T^*) = \Delta Ecorr(T) \cdot \Delta Ecorr(MP2\text{-}F12)/\ \Delta Ecorr(MP2)$$

All calculations were performed using aug-cc-pVTZ-F12 basis sets[11] (cc-pVTZ-F12 for Be[12]) as well as the respective auxiliary basis sets automatically assigned by the MOLPRO program.

Anharmonic vibrational frequencies were calculated at the VCISDTQP6 level of theory allowing up to 5 excitations within one mode.[13] VCI calculations were performed on a polynomial fit of a multi-level surface using CCSD(T*)-F12a/aug-cc-pVDZ-F12 energies for the 2-body and CCSD-F12a/aug-cc-pVDZ-F12 energies for the 3-body terms. Intensities were computed using the dipole surfaces at the HF level of theory.[14]

Vertical excitation energies were calculated at the LR-SCS-CC2[15] and LR-CCSD levels[16] using aug-cc-pVQZ basis sets[17] with the TURBOMOLE program package. Calculations at the LR-CCSDT level[18] used aug-cc-pVDZ basis sets and the MRCC program package.[19] Excitation energies at the LR-CCSDT/aug-cc-pVQZ level are estimated as:

$$\Delta E\ (\text{LR-CCSDT/QZ}) = \Delta E\ (\text{LR-CCSDT/DZ}) + \Delta E\ (\text{LR-CCSD/QZ}) - \Delta E\ (\text{LR-CCSD/DZ})$$

Band splittings according to the Renner-Teller effect were calculated using the expressions (I, 44), (I, 47) and (I,48) provided by Herzberg.[20] The expressions require three input parameters: The vibrational frequency of the bending mode (here taken from our high-level calculations described above), the Renner parameter ε and the spin-orbit coupling strength A. Both ε and A were calculated at the MRCI+Q-F12 level[21] using the basis sets described above. All calculations are based on SA-CASSCF(5,6) calculations using the first two doublet states (the degenerate components of the $^2\Pi$ state). The active space included the bonding and anti-bonding σ- and π- orbitals of the Be-O bond. Inclusion of orbitals on F resulted in rotations with lower-lying orbitals and to natural orbital occupation numbers very close to two. For the calculation of ε, the vibrational frequencies of the bending mode were calculated using either one of the two lowest doublet states at MRCI+Q level. The Renner parameter is then approximately obtained as $\varepsilon \approx (\nu_1^2 - \nu_2^2)/(\nu_1^2 + \nu_2^2)$, where ν_i denotes the vibrational frequency of the bending mode in the *i*th doublet state. Spin-orbit coupling (SOC) eigenstates were obtained by diagonalization of the sum of the electronic and Breit-Pauli SOC Hamiltonian in the basis of the MRCI wave functions obtained for the two doublet states.[22] The energy eigenvalues of the electronic Hamiltonian were adapted to include the Davidson size-consistency correction (+Q)[23] using a relaxed reference. The SOC constant A can then be found as two-thirds of the energy difference between the two eigenstates.

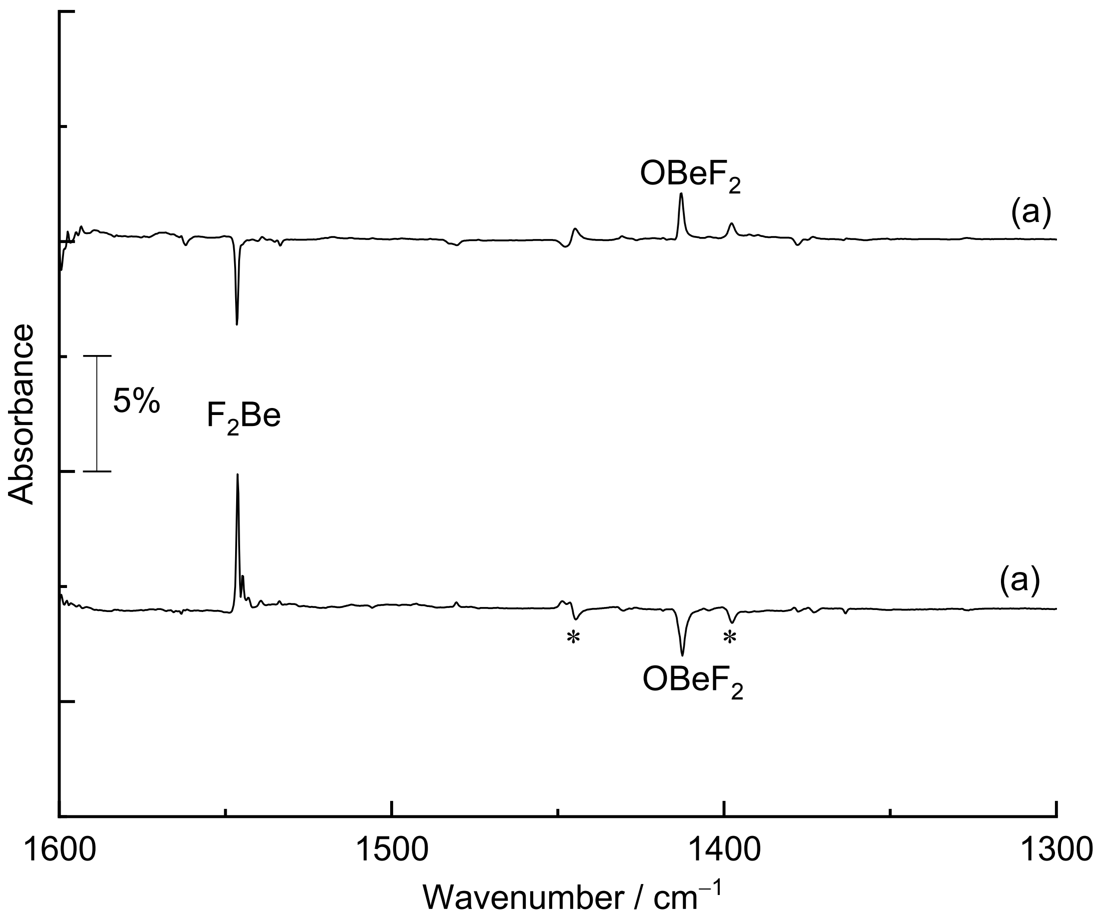

Figure S1. Infrared difference spectra in the 1600–1300 cm^{-1} region from co-deposition of laser-ablated Be atoms with 0.05% OF_2 in neon. (a) Spectrum taken after annealing to 10 K minus the spectrum taken after 30 min of sample deposition at 5 K, (b) Spectrum taken after 10 min of full arc ($\lambda > 220$ nm) irradiation minus the spectrum taken annealing to 10 K. The bands of unidentified species (*) are labeled.

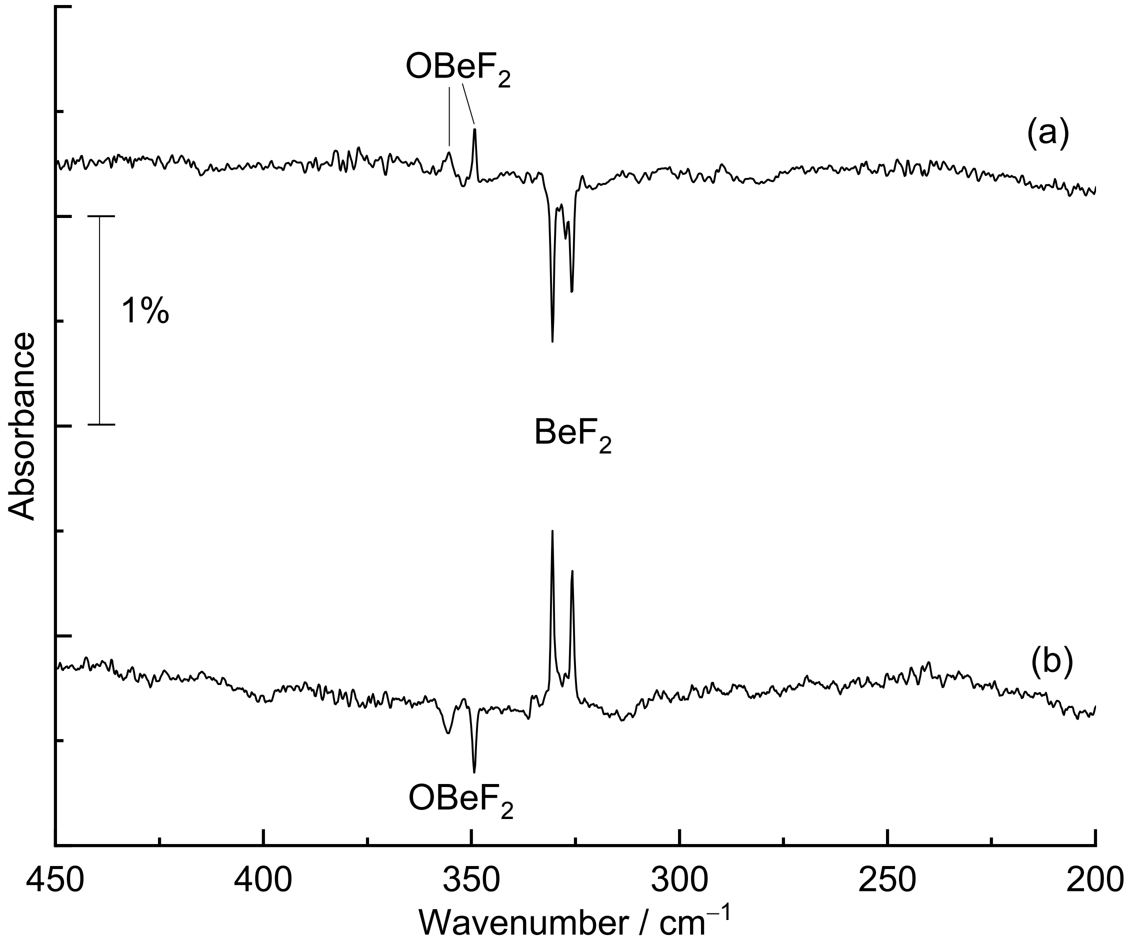

Figure S2. Infrared difference spectra in the 450–200 cm^{-1} region from co-deposition of laser-ablated Be atoms with 0.05% OF_2 in neon. (a) Spectrum taken after annealing to 10 K minus the spectrum taken after 30 min of sample deposition at 5 K, (b) Spectrum taken after 10 min of full arc (λ > 220 nm) irradiation minus the spectrum taken annealing to 10 K.

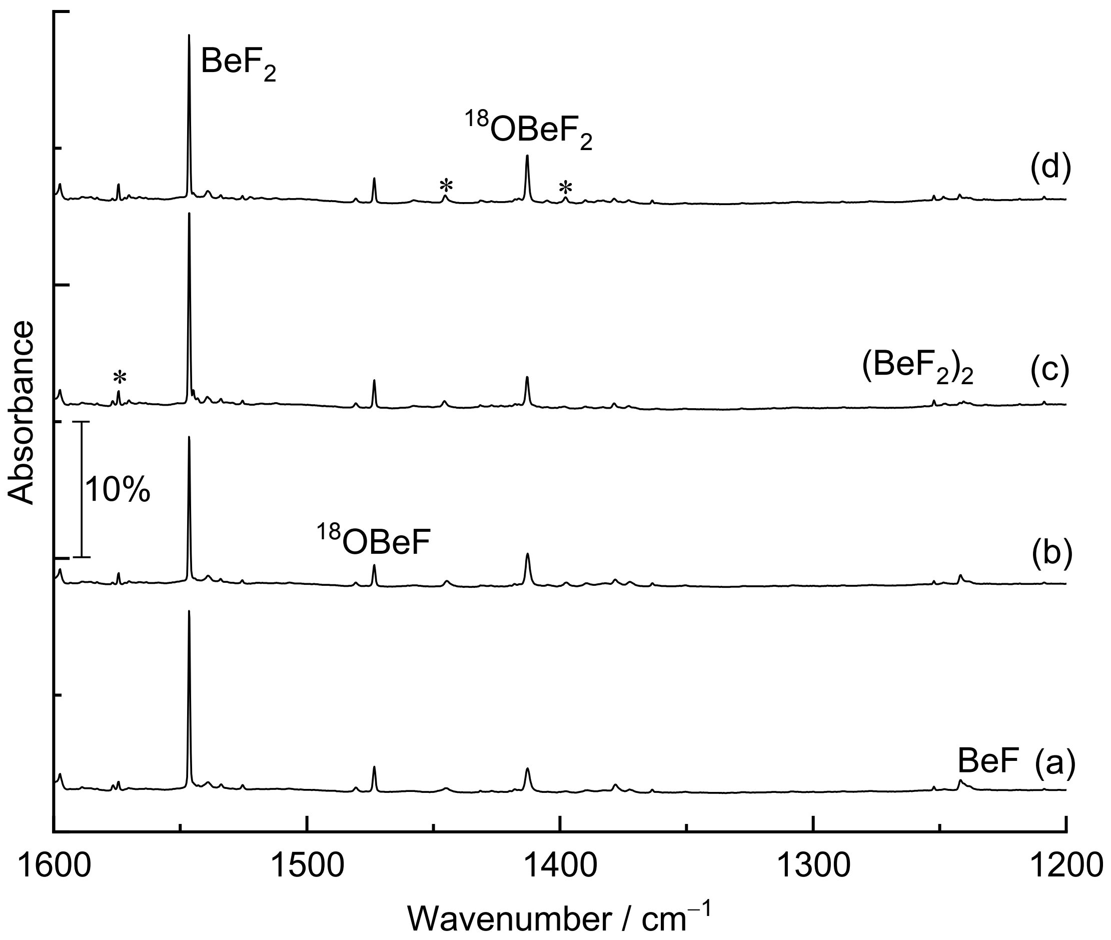

Figure S3. Infrared spectra in the 1600–1200 cm^{-1} region from co-deposition of laser-ablated Be atoms with 0.05% $^{18}OF_2$ in neon. (a) After 30 min of sample deposition, (b) after annealing to 10 K, (c) after 10 min of full arc ($\lambda > 220$ nm) irradiation, (d) after annealing to 10 K. The bands of unidentified species (*) are labeled.

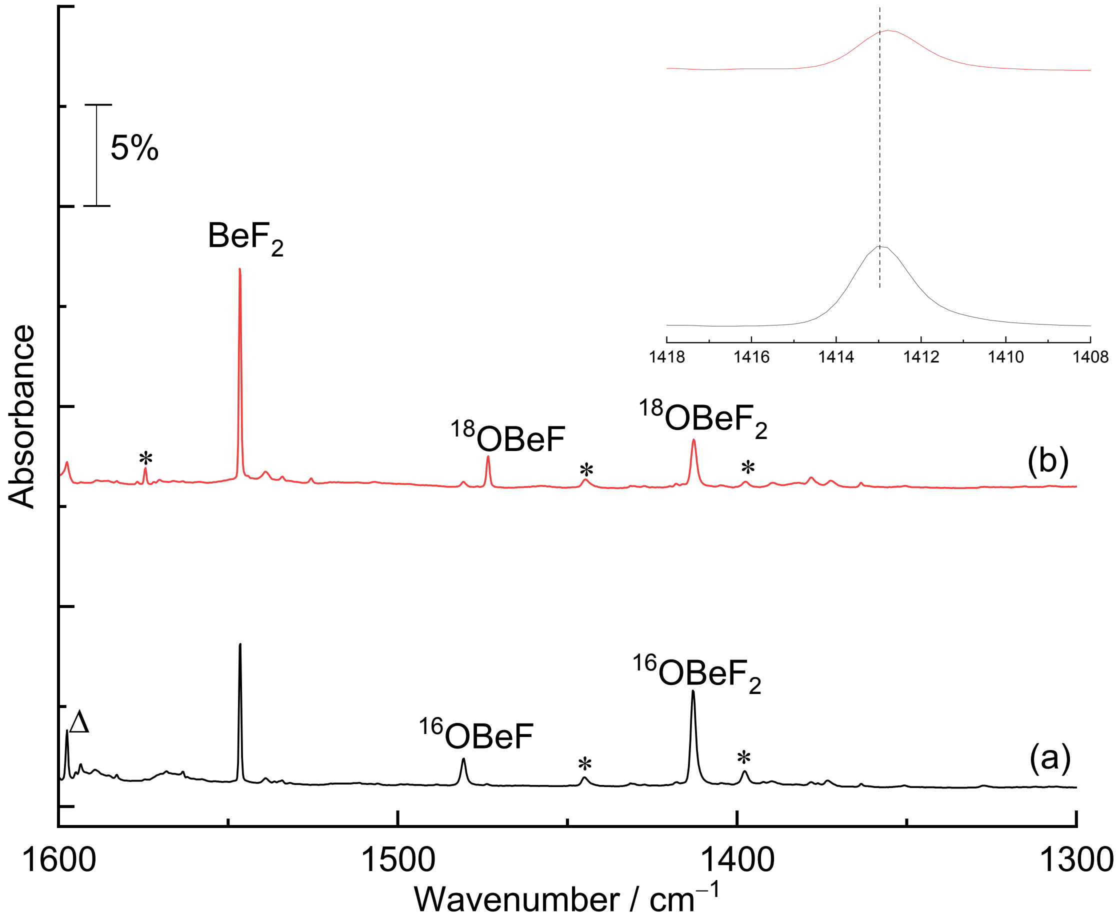

Figure S4. Infrared spectra in the 1600–1300 cm^{-1} region from co-deposition of laser-ablated Be atoms with 0.05% OF_2 in neon after annealing to 10 K. (a) $^{16}OF_2$, (b) $^{18}OF_2$. Inset: Parts of the expanded IR spectra (a) and (b) in the range of 1418–1408 cm^{-1}. The bands of unidentified species (*) are labeled.

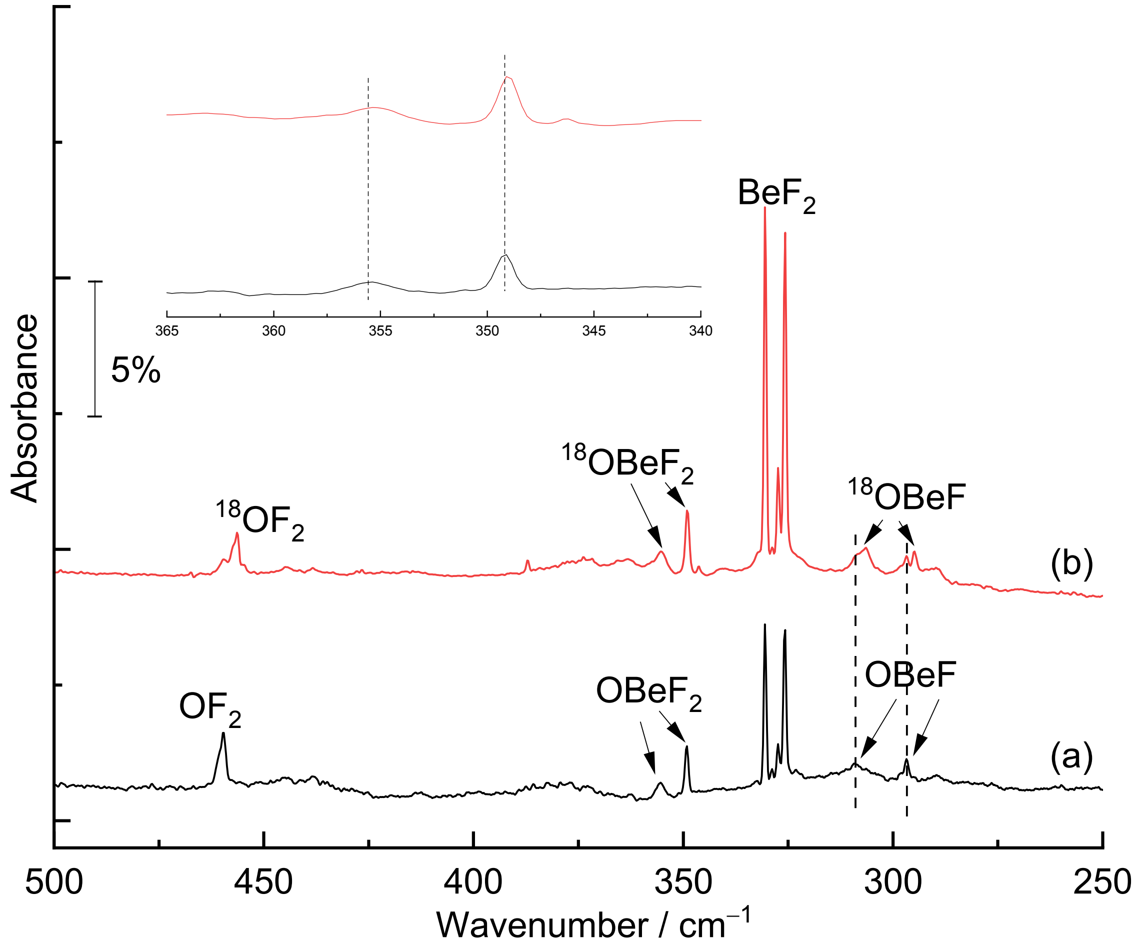

Figure S5. Infrared spectra in the 500–250 cm^{-1} region from co-deposition of laser-ablated Be atoms with 0.05% OF_2 in neon after annealing to 10 K. (a) $^{16}OF_2$, (b) $^{18}OF_2$. Inset: Parts of the expanded IR spectra (a) and (b) in the range of 365–340 cm^{-1}.

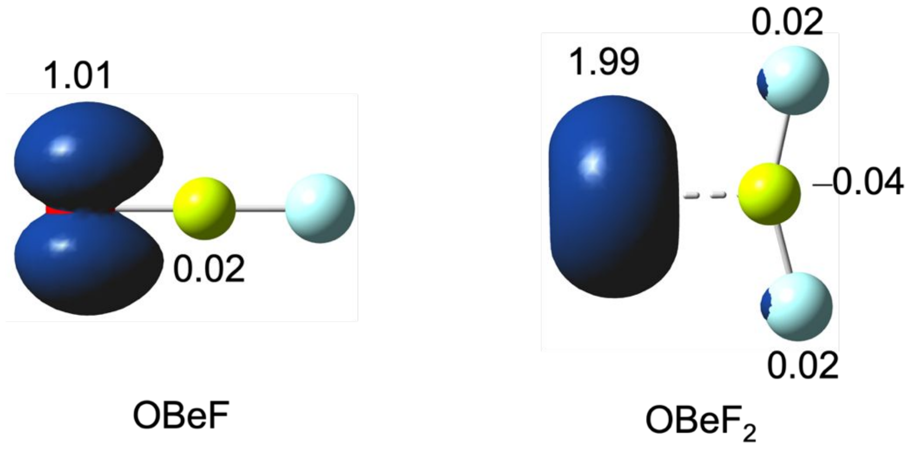

Figure S6. Calculated spin-density for beryllium oxyfluorides at the M06-2X/def2-TZVPP level. Isosurfaces of the densities are shown at a value of 0.005 a.u.

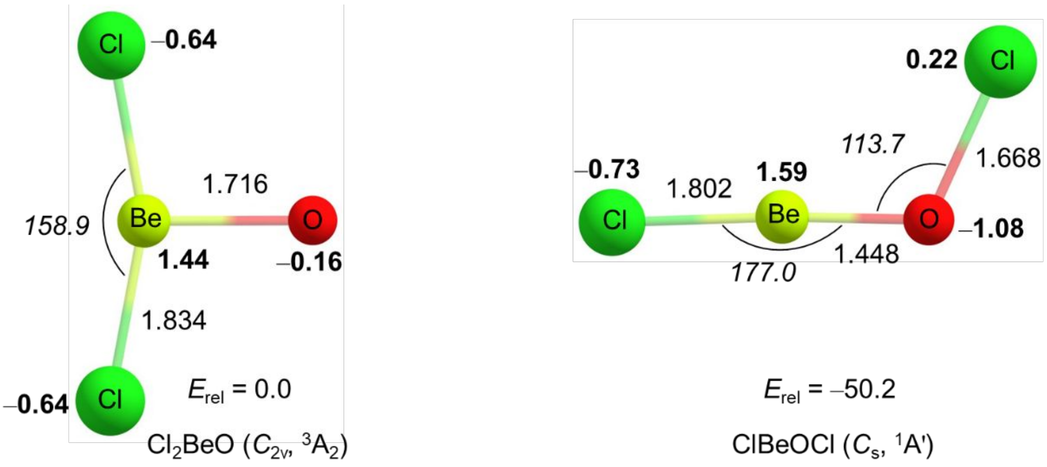

Figure S7. Computed structures (bond lengths in Ångstroms and bond angles in degrees) and NPA charges (in bold) of beryllium oxychlorides at the M06-2X/def2-TZVPP level.

Table S1. Experimentally observed and calculated vibrational frequencies (cm^{-1}) and intensities ($km\,mol^{-1}$) of $OBeF_2$ (M06-2X/def2-TZVPP, CCSD(T)/aug-cc-pVTZ and CCSD(T*)-F12a/(aug-)cc-pVTZ-F12 levels).

Exptl		Calcd					
		M06-2X		CCSD(T)		CCSD(T*)-F12a [a]	
ν(^{16}O)	Δν($^{16/18}$O)	ν	Δν	N	Δν	N	Δν
1413.1	0.2	1457.8 (342)	0.0	1434.1	0.0	1419.9 (405)	0.1
		774.4 (83)	0.1	760.0	0.1	787.4 (42)	3.8
355.5	0.6	378.2 (145)	1.0	370.7	0.9	355.6 (175)	0.2
349.3	0.3	350.0 (146)	0.1	358.5	0.3	349.8 (197)	0.4
		257.1 (7)	0.3	245.8	0.0	229.2 (<1)	9.0
		43 (<1)	1.0	125.5	3.5	129.8 (<1)	3.0

[a] Anharmonic vibrational frequencies obtained at VCISDTQP6 level. Intensities were obtained based on dipole surfaces at the HF level.

Table S2. Experimentally observed and calculated vibrational frequencies (cm^{-1}) and intensities ($km\,mol^{-1}$) of OBeF (M06-2X/def2-TZVPP, CCSD(T)/aug-cc-pVTZ and CCSD(T*)-F12a/(aug-)cc-pVTZ-F12 levels).

Exptl		Calcd					
		M06-2X[b][c]		CCSD(T)[c]		CCSD(T*)-F12a [a]	
ν(^{16}O)	Δν($^{16/18}$O)	ν(^{16}O)	Δν	ν(^{16}O)	Δν	ν(^{16}O)	Δν
1480.6	7.2	1531.0 (339)	7.0	1495.9	6.6	1476.7 (318)	7.7
		719.7 (<1)	22.7	700.3	22.3	726.3 (<1)	18.2
309.3	2.8	314.8 (158)	1.8	311.5	1.8	306.7 (193)	1.8
296.7	1.7	312.8 (158)	1.8	309.0	1.8	306.7 (193)	1.8

[a] Anharmonic vibrational frequencies obtained at VCISDTQP6 level. Intensities were obtained based on dipole surfaces at the HF level. [b] Frequencies have been calculated using the NumForce script of TURBOMOLE. [c] The remaining difference between the two degenerate modes is due to limited numerical precision.

Table S3. Calculated vibrational frequencies (cm^{-1}) and intensities ($km\ mol^{-1}$) of FOBeF at the M06-2X/def2-TZVPP, CCSD(T)/aug-cc-pVTZ, and CCSD(T*)-F12a/(aug-)cc-pVTZ-F12 levels.

M06-2X		CCSD(T)		CCSD(T*)-F12a[a]	
$\nu(^{16}O)$	$\Delta\nu(^{16/18}O)$	$\nu(^{16}O)$	$\Delta\nu(^{16/18}O)$	$\nu(^{16}O)$	$\Delta\nu(^{16/18}O)$
1478.7 (403)	3.6	1438.3	4.5	1426.9 (269)	6.6
931.0 (43)	25.0	807.6	22.0	798.0 (100)	18.9
676.4 (12)	17.8	653.7	16.3	614.7 (12)	10.4
378.7 (78)	7.6	411.3	3.0	392.5 (53)	3.0
335.9 (133)	2.2	328.4	1.0	332.2 (164)	1.1
244.5 (24)	1.0	242.5	2.3	240.4 (38)	2.3

[a] Anharmonic vibrational frequencies obtained at VCISDTQP6 level. Intensities were obtained based on dipole surfaces at the HF level.

Table S4. Vertical excitation energies (in eV) and oscillator strengths of $OBeF_2$ at various coupled-cluster levels (see above for details).

Excitations	ΔE^a	oscillator strength^a	ΔE^b
1 A_1	6.35	0.02643845	5.69
2 A_1	6.81	0.01393970	6.17
3 A_1	8.17	0.00925296	7.46
1 B_1	0.93	0.00000541	0.80
2 B_1	6.15	0.00614351	5.53
3 B_1	6.91	0.00841868	6.27
1 B_2	0.70	0.00003908	0.58
2 B_2	6.40	0.00070029	5.75
3 B_2	6.64	0.00007644	6.05

^{a}Calculated at the LR-SCS-CC2/aug-cc-pVQZ level. ^{b}Calculated at an approximate LR-CCSDT/aug-cc-pVQZ level (see above for details).

Table S5. Experimentally observed and calculated vibrational frequencies (cm^{-1}) and intensities ($km\ mol^{-1}$) of FBeONe (CCSD(T*)-F12a/(aug-)cc-pVTZ-F12 level).

Exptl		Calcd		Assignment
		CCSD(T*)-F12a [a]		
$\nu(^{16}O)$	$\Delta\nu(^{16/18}O)$	$\nu(^{16}O)$	$\Delta\nu$	
1480.6	7.2	1475.4 (156)	5.8	O-Be–F asymmetric str.
--	--	725.5 (<1)	16.4	O-Be-F symmetric str.
309.3	2.8	330 (80)	5.3	OBeF in-plane bending
296.7	1.7	298.3 (205)	1.4	OBeF out-of-plane bending
--	--	49.5 (<1)	2.2	Ne-Be str.
--	--	33.2 (<1)	0.5	NeBeF in plane bending

[a] Anharmonic vibrational frequencies obtained at VCISDTQP6 level. Intensities were obtained based on dipole surfaces at the HF level.

Table S6. Calculated rovibronic transitions due to the Renner-Teller effect in OBeF using the parameters $|\varepsilon| = 0.104$ and $|A| = 82\ cm^{-1}$ mentioned above.

Transition	$\Delta E\ [cm^{-1}]$
$^2\Pi_{1/2} \longrightarrow {}^2\Pi_{3/2}$	82
$^2\Pi_{1/2} \longrightarrow {}^2\Sigma_{1/2}$	295
$^2\Pi_{1/2} \longrightarrow {}^2\Delta_{3/2}$	306
$^2\Pi_{1/2} \longrightarrow {}^2\Delta_{5/2}$	387
$^2\Pi_{1/2} \longrightarrow {}^2\Sigma_{1/2}$	399

Table S7. Calculated harmonic vibrational frequencies (in cm^{-1}) of OBeF at different levels of theory.

	CCSD(T*)-F12a[a]	CCSD(T*)-F12a[b]	MRCI+Q-F12[b]
ν (1 A_1)	1503.5	1503.5	1513.9
ν (2 A_1)	705.2	705.2	709.1
ν (E (1))	336.0	309.0	305.3
ν (E (2))	308.4	308.4	304.2

[a]Results obtained by using symmetry to ensure the same state during all displacements. [b]Results obtained by using no symmetry throughout the calculation.

Table S8. NPA charges (M06-2X/def2-TZVPP) and EDA results (BP86+D3(BJ)/TZ2P) for the Be–X bond in BeX_2 (X = F, Cl).

	X = F	X = Cl
q^{Be} (BeX_2) [e]	1.736	1.450
ΔE_{Pauli} (X–BeX) [kcal mol^{-1}]	227.0	183.6
ΔE_{elstat} (X–BeX) [kcal mol^{-1}]	–101.9	–122.0
$\Delta E_{orbital}$ (X–BeX) [kcal mol^{-1}]	–293.8	–188.4
ΔE_{Total} (X–BeX) [kcal mol^{-1}]	–169.0	–128.1

Table S9. Bond dissociation energies (in kcal mol^{-1}) at the CCSD(T*)-F12a/(aug-)cc-pVTZ-F12 level. Data do not include nuclear relaxation of dissociated diatomic fragments.

	X = F	X = Cl
BDE (X–BeX)	170.9	132.0
BDE (X–Be(O)X)	177.0	138.2
BDE (X–BeOX)	173.7	129.9
BDE (XBe–OX)	132.0	123.3

References

[1] Schlöder, T.; Vent-Schmidt, T.; Riedel, S. A Matrix-Isolation and Quantum-Chemical Investigation of FeF_4. *Angew. Chem., Int. Ed.* **2012**, *51*, 12063– 12067.

[2] Borning, A. H.; Pullen, K. E. A Simple Preparation of Oxygen Difluoride in High Yield. *Inorg. Chem.* **1969**, *8*, 1791.

[3] a) Zhao, Y.; Truhlar, D. G. The M06 suite of density functionals for main group thermochemistry, thermochemical kinetics, noncovalent interactions, excited states, and transition elements: two new functionals and systematic testing of four M06-class functionals and 12 other functionals. *Theor. Chem. Acc.* **2008**, *120*, 215–241; b) Weigend, F.; Ahlrichs, R. Balanced basis sets of split valence, triple zeta valence and quadruple zeta valence quality for H to Rn: Design and assessment of accuracy. *Phys. Chem. Chem. Phys.* **2005**, *7*, 3297–3305.

[4] Frisch, M. J.; Trucks, G. W.; Schlegel, H. B.; Scuseria, G. E.; Robb, M. A.; Cheeseman, J. R.; Scalmani, G.; Barone, V.; Petersson, G. A.; Nakatsuji, H.; Li, X.; Caricato, M.; Marenich, A. V.; Bloino, J.; Janesko, B. G.; Gomperts, R.; Mennucci, B.; Hratchian, H. P.; Ortiz, J. V.; Izmaylov, A. F.; Sonnenberg, J. L.; Williams-Young, D.; Ding, F.; Lipparini, F.; Egidi, F.; Goings, J.; Peng, B.; Petrone, A.; Henderson, T.; Ranasinghe, D.; Zakrzewski, V. G.; Gao, J.; Rega, N.; Zheng, G.; Liang, W.; Hada, M.; Ehara, M.; Toyota, K.; Fukuda, R.; Hasegawa, J.; Ishida, M.; Nakajima, T.; Honda, Y.; Kitao, O.; Nakai, H.; Vreven, T.; Throssell, K.; Montgomery, J. A., Jr.; Peralta, J. E.; Ogliaro, F.; Bearpark, M. J.; Heyd, J. J.; Brothers, E. N.; Kudin, K. N.; Staroverov, V. N.; Keith, T. A.; Kobayashi, R.; Normand, J.; Raghavachari, K.; Rendell, A. P.; Burant, J. C.; Iyengar, S. S.; Tomasi, J.; Cossi, M.; Millam, J. M.; Klene, M.; Adamo, C.; Cammi, R.; Ochterski, J. W.; Martin, R. L.; Morokuma, K.; Farkas, O.; Foresman, J. B.; Fox, D. J. *Gaussian 16*, revision C.01; Gaussian, Inc.: Wallingford, CT, **2016**.

[5] a) Reed, A. E.; Curtiss, L. A.; Weinhold, F. Intermolecular Interactions from a Natural Bond Orbital, Donor-Acceptor Viewpoint. *Chem. Rev.* **1988**, *88*, 899–926; b) Reed, A. E.; Weinstock, R. B.; Weinhold, F. Natural Population Analysis. *J. Chem. Phys.* **1985**, *83*, 735–746.

[6] Franzke, Y. J.; Holzer, C.; Andersen, J. H.; Begušić, T.; Bruder, F.; Coriani, S.; Della Sala, F.; Fabiano, E.; Fedotov, D. A.; Fürst, S; Gillhuber, S.; Grotjahn, R.; Kaupp, M.; Kehry, M.; Krstić, M.; Mack, F.; Majumdar, S.; Nguyen, B. D.; Parker, S. M.; Pauly, F.; Pausch, A.; Perlt, E.; Phun, G. S.; Rajabi, A.; Rappoport, D.; Samal, B.; Schrader, T.; Sharma, M.; Tapavicza, E.; Treß, R. S.; Voora, V.; Wodyński, A.; Yu, J. M.; Zerulla, B.; Furche, F.; Hättig, C.; Sierka, M.; Tew, D. P.; Weigend, F. TURBOMOLE: Today and Tomorrow. *J. Chem. Theory Comput.* **2023**, *19*, 6859–6890.

[7] a) Becke, A. D. Density-functional exchange-energy approximation with correct asymptotic behavior. *Phys. Rev. B* **1988**, *38*, 3098–3100; b) Perdew, J. P. Density-Functional Approximation for the Correlation Energy of the Inhomogeneous Electron Gas. *Phys. Rev. B: Condens. Matter Mater. Phys.* **1986**, *33*, 8822–8824; c) Perdew, J. P. Erratum: Density-Functional Approximation for the Correlation Energy of the Inhomogeneous Electron Gas. *Phys. Rev. B: Condens. Matter Mater. Phys*. **1986**, *34*, 7406; d) Grimme, S.; Ehrlich, S.; Goerigk, L. Effect of the Damping Function in Dispersion Corrected Density Functional Theory. *J. Comput. Chem.* **2011**, *32*, 1456–1465; e) Van Lenthe, E.; Baerends, E. J. Optimized Slater-Type Basis Sets for the Elements 1–118. *J. Comput. Chem*. **2003**, *24*, 1142–1156.

[8] a) te Velde, G.; Bickelhaupt, F. M.; Baerends, E. J.; Fonseca Guerra, C.; van Gisbergen, S. J. A.; Snijders, J. G.; Ziegler, T. Chemistry with ADF. *J. Comput. Chem*. **2001**, *22*, 931– 967; b) Rüger, R.; Franchini, M.; Trnka, T.; Yakovlev, A.; van Lenthe, E.; Philipsen, P.; van Vuren, T.; Klumpers, B.; Soini, T.; AMS 2023.1, SCM, Theoretical Chemistry, Vrije Universiteit, Amsterdam, The Netherlands, https://www.scm.com.

[9] Werner, H.-J.; Knowles, P. J.; Celani, P.; Györffy, W.; Hesselmann, A.; Kats, D.; Knizia, G.; Köhn, A.; Korona, T.; Kreplin, D.; Lindh, R.; Ma, Q.; Manby, F. R.; Mitrushenkov, A.; Rauhut, G.; Schütz, M.; Shamasundar, K. R.; Adler, T. B.; Amos, R. D.; Bennie, S. J.; Bernhardsson, A.; Berning, A.; Black, J. A.; Bygrave, P. J.; Cimiraglia, R.; Cooper, D. L.; Coughtrie, D.; Deegan, M. J. O.; Dobbyn, A. J.; Doll, K.; Dornbach, M.; Eckert, F.; Erfort, S.; Goll, E.; Hampel, C.; Hetzer, G.; Hill, J. G.; Hodges, M.; Hrenar, T.; Jansen, G.; Köppl, C.; Kollmar, C.; Lee, S. J. R.; Liu, Y.; Lloyd, A. W.; Mata, R. A.; May, A. J.; Mussard, B.; McNicholas, S. J.; Meyer, W.; Miller, III, T. F.; Mura, M. E.; Nicklass, A.; O'Neill, D. P.; Palmieri, P.; Peng, D.; Peterson, K. A.; Pflüger, K.; Pitzer, R.; Polyak, I.; Reiher, M.; Richardson, J. O.; Robinson, J. B.; Schröder, B.; Schwilk, M.; Shiozaki, T.; Sibaev, M.; Stoll, H.; Stone, A. J.; Tarroni, R.; Thorsteinsson, T.; Toulouse, J.; Wang, M.; Welborn, M.; Ziegler, B. *MOLPRO*, version 2022.2, a package of ab initio programs; Cardiff, U. K. **2022**, see https://www.MOLPRO.net.

[10] a) Knizia, G.; Adler, T. B.; Werner, H.-J. Simplified CCSD(T)-F12 Methods: Theory and Benchmarks. *J. Chem. Phys*. **2009**, *130*, 54104; b) Adler, T. B.; Knizia, G.; Werner, H.-J. A Simple and Efficient CCSD(T)-F12 Approximation. *J. Chem. Phys*. **2007**, *127*, 221106.

[11] Sylvetsky, N.; Kesharwani, M. K.; Martin, J. M. L. The aug-cc-pVnZ-F12 Basis Set Family: Correlation Consistent Basis Sets for Explicitly Correlated Benchmark Calculations on Anions and Noncovalent Complexes. *J. Chem. Phys*. **2017**, *147*, 134106.

[12] Peterson, K. A.; Adler, T. B.; Werner, H.-J. Systematically convergent basis sets for explicitly correlated wavefunctions: The atoms H, He, B–Ne, and Al–Ar. *J. Chem. Phys*. **2008**, *128*, 084102.
[13] a) Mathea, T.; Petrenko, T.; Rauhut, G. Advances in Vibrational Configuration Interaction Theory – part 2: Fast Screening of the Correlation Space. *J. Comput. Chem*. **2022**, *43*, 6–18; b) Mathea, T.; Rauhut, G. Advances in Vibrational Configuration Interaction Theory-part 1: Efficient Calculation of Vibrational Aangular Momentum Terms. *J. Comput. Chem*. **2021**, *42*, 2321–2333.
[14] a) Ziegler, B.; Rauhut, G. Efficient Generation of Sum-of-products Representations of High-dimensional Potential Energy Surfaces Based on Multimode Expansions. *J. Chem. Phys*. **2016**, *144*, 114114; b) Rauhut, G. Efficient Calculation of Potential Energy Surfaces for the Generation of Vibrational Wave Functions. *J. Chem. Phys*. **2004**, *121*, 9313–9322.
[15] Hellweg, A.; Gruan, S.; Hättig, C. Benchmarking the Performance of Spin-Component Scaled CC2 in Ground and Electronically Excited States. *Phys. Chem. Chem. Phys*. **2008**, *10*, 4119–4127.
[16] Purvis, G. D.; Bartlett, R. J. A. Full Coupled-Cluster Singles and Doubles Model: The Inclusion of Disconnected Triples. *J. Chem. Phys.* **1982**, *76*, 1910–1918.
[17] Kendall, R. A.; Dunning, T. H.; Harrison, R. J. Electron affinities of the first-row atoms revisited. Systematic basis sets and wave functions. *J. Chem. Phys.* **1992**, *96*, 6796–6806.
[18] Piecuch, P.; Kucharski, S. A.; Bartlett, R. J. *J. Chem. Phys.* **1999**, *110*, 6103.
[19] Kállay, M.; Nagy, P. R.; Mester, D.; Rolik, Z.; Samu, G.; Csontos, J.; Csóka, J.; Szabó, P. B.; Gyevi-Nagy, L.; Hégely, B.; Ladjánszki, I.; Szegedy, L.; Ladóczki, B.; Petrov, K.; Farkas, M.; Mezei, P.; Ganyecz, Á. The MRCC Program System: Accurate Quantum Chemistry From Water to Proteins. *J. Chem. Phys.* **2020**, *152*, 074107.
[20] G. Herzberg, Molecular Spectra and Molecular Structure: III. Electronic Spectra and Electronic Structure of Polyatomic Molecules, Van Nostrand Reinhold, New York, **1966**, 27–37.
[21] a) Shiozaki, T.; Knizia, G.; Werner, H.-J. Explicitly Correlated Multireference Configuration Interaction: MRCI-F12. *J. Chem. Phys.* **2011**, *134*, 034113; b) Shiozaki, T.; Werner, H.-J. Multireference Explicitly Correlated F12 Theories. *Mol. Phys*. **2013**, *111*, 607.
[22] Berning, A.; Schweizer, M.; Werner, H. J.; Knowles, P. J.; Palmieri, P. Spin-orbit Matrix Elements for Internally Contracted Multireference Configuration Interaction Wavefunctions. *Mol. Phys.* **2000**, *98*, 1823−1833.

[23] Langhoff, S. R.; Davidson, E. R. Configuration Interaction Calculations on the Nitrogen Molecule. *Int. J. Quantum Chem*. **1974**, *8*, 61–72.

Calculated atomic coordinates (in Å) of species for optimized structures at the M06-2X/def2-TZVPP level.

OBeF ($C_{\infty v}$, $^2\Pi$)

Be	0.00000000	0.00000000	-0.02911600
F	0.00000000	0.00000000	1.34673400
O	0.00000000	0.00000000	-1.50051800

OBeF$_2$ (C_{2v}, $^3A_2$)

Be	0.00000000	0.00000000	-0.30561800
F	0.00000000	1.36065100	-0.62590700
F	0.00000000	-1.36065100	-0.62590700
O	0.00000000	0.00000000	1.56109900

FOBeF (C_s, $^1A'$)

Be	0.00000000	0.40452100	0.00000000
O	-1.08352000	-0.63435600	0.00000000
F	0.28190600	-1.22049000	0.00000000
F	0.68122300	1.60457400	0.00000000

OBeCl$_2$ (C_{2v}, $^3A_2$)

Be	0.00000000	0.00000000	-0.04968500
O	0.00000000	0.00000000	1.66581500
Cl	0.00000000	1.80310500	-0.38611100
Cl	0.00000000	-1.80310500	-0.38611100

ClOBeCl (C_s, $^1A'$)

Be	0.00000000	0.64628500	0.00000000
O	-1.18812600	-0.18167700	0.00000000
Cl	1.42345700	1.75179300	0.00000000
Cl	-0.86433900	-1.81836500	0.00000000

Calculated atomic coordinates (in Å) of species for optimized structures at the CCSD(T*)-F12a/aug-cc-pVTZ-F12 level.

OBeF ($C_{\infty v}$, $^2\Pi$)

Be	0.00000000	0.00000000	0.05700416
O	0.00000000	0.00000000	1.53583463
F	0.00000000	0.00000000	-1.32007266

OBeFNe (C_s, $^1A'$)

Be	0.00000000	-0.20223473	0.86921563
O	0.00000000	-1.62654982	0.47105876
F	0.00000000	1.12026331	1.25414361
Ne	0.00000000	0.32791811	-1.96048068

$OBeF_2$ (C_{2v}, $^3A_2$)

Be	0.00000000	0.00000000	-0.29142966
O	0.00000000	0.00000000	1.61198280
F	0.00000000	1.36348193	-0.60944877
F	0.00000000	-1.36348193	-0.60944877

FOBeF (C_s, $^1A'$)

Be	0.00000000	0.14743086	0.35320968
F	0.00000000	-0.04465356	1.72226176
O	0.00000000	0.81996760	-1.00569333
F	0.00000000	-0.71562001	-1.04311012
